# Accelerated response and reduced chemotherapy with ruxolitinib-based regimen therapy in pediatric hemophagocytic lymphohistiocytosis: a retrospective comparison with HLH-94 regimen

**DOI:** 10.3389/fimmu.2026.1769006

**Published:** 2026-03-26

**Authors:** Na Song, Haiyan Luo, Benshan Zhang, Pan Wu, Xiaoqin Zhang, Qianxiu Fan, Huimin Xu, Zilin Gong, Chenli Zhou, Shiyi Hu, Xinping Zhang, Jiaotian Huang, Haixia Yang

**Affiliations:** 1 Department of Hematology, The Affiliated Children’s Hospital of Xiangya School of Medicine, Central South University (Hunan Children’s Hospital), Changsha, China; 2 Department of Oncology, The Affiliated Children’s Hospital of Xiangya School of Medicine, Central South University (Hunan Children’s Hospital), Changsha, China; 3 Department of Critical Care Medicine, The Affiliated Children’s Hospital of Xiangya School of Medicine, Central South University (Hunan Children’s Hospital), Changsha, China

**Keywords:** chemotherapy reduction, children, efficacy, hemophagocytic lymphohistiocytosis, retrospective study, ruxolitinib

## Abstract

**Objective:**

To compare the efficacy of a Ruxolitinib-based regimen versus the conventional HLH-94 protocol in children with newly diagnosed hemophagocytic lymphohistiocytosis (HLH).

**Methods:**

This single-center, retrospective historical control study enrolled 67 pediatric HLH patients who met the HLH-2004 diagnostic criteria. Patients treated with the HLH-94 regimen (dexamethasone plus etoposide) from 2022 to 2023 constituted the control group (Group C, n=40), while those treated with a response-guided, ruxolitinib-based regimen from 2024 comprised the treatment group (Group T, n=27). Primary endpoint was 12-month overall survival (OS). Secondary endpoints included early response, safety, and cumulative exposure to glucocorticoids and etoposide during the first 8 weeks of therapy.

**Results:**

Group T achieved a significantly higher complete response (CR) rate at week 2 compared to Group C (25.9% vs. 0%; P = 0.001). CR rates at weeks 4 and 8 were 55.5% vs. 35.0% (P = 0.096) and 78.0% vs. 70.0% (P = 0.481), respectively, with no significant difference in overall response rate. The 12-month OS (96.3% vs. 92.5%) and EFS (74.1% vs. 77.5%) rates did not differ significantly (P>0.05). Serial laboratory monitoring revealed that Group T exhibited faster hematological recovery and earlier neutrophil normalization (by week 2 vs. week 4) (P < 0.001). Key inflammatory markers (ferritin, IFN-γ, IL-10) declined rapidly in both groups. Crucially, the ruxolitinib-based strategy drastically reduced chemotherapy exposure: a significantly lower proportion of patients in Group T received glucocorticoids (66.7% vs. 100%, P<0.001) and etoposide (41.7% vs. 100%, P<0.001), and a significantly different distribution of glucocorticoids(Group T median: 60mg/kg (IQR: 0, 60); Group C median: 60mg/kg (IQR:60, 60); P = 0.012)and etoposide(Group T median: 0mg/m2 (IQR: 0, 885.5mg/m2); Group C median: 900mg/m2 (IQR:900, 1000); p=0.000) The incidence of secondary infections and treatment-related laboratory abnormalities (TBil, AST, ALT, or Scr) was comparable between groups.

**Conclusion:**

For pediatric HLH, a ruxolitinib-based regimen is a viable and effective therapy. It facilitates more rapid complete remission and markedly reduces chemotherapy exposure, particularly to etoposide, while preserving high short-term survival with an acceptable safety profile. This study provides a compelling rationale for future prospective randomized controlled trials to confirm the long-term benefits and safety of this approach.

## Introduction

Hemophagocytic lymphohistiocytosis (HLH) is a lethal disorder characterized by pathologic immune activation and hyperinflammation. Based on etiology, HLH can be classified into primary HLH and secondary HLH. Etoposide-based HLH-1994 regimens remain widely accepted as the standard of treatment, substantially improving survival in patients with this fatal condition. Despite this, 30%˜40% of HLH patients fail to respond to conventional therapy, with mortality rates remaining as high as 40% (1, 2). Furthermore, etoposide’s myelosuppressive effects and high-dose corticosteroid toxicities frequently cause severe adverse events (AEs) (3), including sepsis, hepatic/renal impairment, and secondary malignancies (4), particularly problematic in pediatric populations. This toxicity profile underscores the urgent need for targeted, less cytotoxic alternatives.

Recent advances highlight Janus kinase (JAK) 1/2 inhibition as a promising strategy. Ruxolitinib, an oral JAK1/2 inhibitor, selectively blocks signaling pathways of key HLH-associated cytokines, including interferon-γ (IFN-γ), interleukin-6 (IL-6), and interleukin-2 (IL-2) (5, 6). Data from HLH animal models have confirmed that this small molecule is a suitable agent for HLH treatment (6–8). Clinical evidence demonstrates its capacity to rapidly alleviate core HLH manifestations—normalizing fever within 48 hours, resolving cytopenias, and reducing hyperferritinemia and hepatic dysfunction—while exhibiting favorable tolerability in children. Notably, retrospective and pilot prospective studies in pediatric HLH populations report response rates of 83.3%-96.2% with ruxolitinib-based regimens, including complete response (CR) rates of 66.7%-92.3% (9–11). Furthermore, ruxolitinib enables significant glucocorticoid dose reduction (≥50% cumulative decrease), mitigating steroid-related AEs (12).

Building on these findings, response-based stratified approaches integrating ruxolitinib have emerged. These strategies tailor therapy intensity: favorable response patients may receive ruxolitinib monotherapy, while unfavorable response cases combine ruxolitinib with reduced-dose or deferred etoposide (9, 11, 13). Such regimens potentially minimize chemotherapy exposure—observational data indicate up to 38.1% of pediatric primary HLH patients avoided etoposide entirely when treated with ruxolitinib-based protocols (9). However, key evidence gaps remain: direct comparative data evaluating ruxolitinib-based regimens versus conventional chemotherapy are scarce.

This retrospective study aimed to evaluate the treatment response rate and survival outcomes of a ruxolitinib-based regimen in pediatric HLH compared to the conventional chemotherapy protocol, thereby providing evidence-based support for clinical decision-making.

## Method

### Study design and patients

This retrospective historical control study analyzed the response and outcomes of pediatric hemophagocytic lymphohistiocytosis (HLH) patients. Patients receiving a Ruxolitinib-based regimen from January 2024 to December 2024 at Hunan Children’s Hospital were compared with historical controls receiving conventional chemotherapeutic regimens (HLH-94 protocols) treated from January 2022 to December 2023 at the same institution. Patients were consecutively enrolled. Data were obtained from real-world clinical records. The study adhered to the principles of the Declaration of Helsinki and received approval from the Institutional Review Board of Hunan Children’s Hospital(KS2025-269). The informed consent was exempted from the patients or their parents.

Patients enrolled in this study fulfilled the following criteria: (1) Met the HLH-2004 diagnostic criteria (14, 15); (2) Had a new diagnosis of HLH; (3) Were aged less than 18 years. (4) Completed the full 8-week course of first-line therapy for HLH. Patients were excluded if they met any of the following conditions: (1) Concurrent malignancy; (2) Discontinuation due to patient or family request during the course of treatment.

### Treatment and assessment

Patients were stratified into two cohorts based on the time period and the corresponding institutional protocol. Group T received Ruxolitinib-based regimen (implemented 2024). Group C received the conventional chemotherapy regimen HLH-94 (administered 2022-2023), consisting of dexamethasone and etoposide. Ruxolitinib-based regimen of Group T: (1) frontline treatment comprised oral ruxolitinib, with doses adjusted by body weight: 2.5 mg twice daily for patients ≤10 kg, 5 mg twice daily for patients ≤20 kg, and 10 mg twice daily for patients >20 kg. (2) The following treatment course based on response: (i) Favorable Response: Patients continued ruxolitinib monotherapy for a total treatment duration of 8 weeks. (ii) Unfavorable Response: Additional chemotherapy for HLH was added and continued for 8 weeks. Options included methylprednisolone (MP) alone or methylprednisolone (MP) plus etoposide. Additional therapy was individualized based on patient response and clinical assessments. For patients presenting with hypotension, organ dysfunction, or central nervous system (CNS) involvement, administration of MP and etoposide could be intensified. Definition of unfavorable response: no response after 3 days of treatment; disease improvement without achieving a partial response after 1 week; or disease progression at any time during front-line therapy. For all patients who achieved no remission, experienced disease progression, or relapsed, salvage therapy with the ruxolitinib plus DEP/L-DEP regimen was administered to serve as a bridge to hematopoietic stem cell transplantation (HSCT).

Evaluations of treatment response were performed on week 2, 4 and 8 after treatment. Overall response rate (ORR) was defined as the proportion of patients with complete response (CR), partial response (PR) and HLH improvement. CR was defined as the normalization of all of the quantifiable symptoms and laboratory markers of HLH (13): (1)No fever: body temperature <37.5 °C;(2)Normal spleen size measured by abdominal ultrasound; (3)No cytopenia in the absence of transfusion support; (4) fibrinogen levels >1.50 g/L; (5) Normal levels of soluble CD25, ferritin, triglyceride and HLH key cytokines including INF-γ and IL-10; (6)EBV DNA in plasma had to revert to negative for EBV-HLH; (7)No central nervous system (CNS) abnormalities for CNS involvement. PR was defined as meeting all the following criteria (13): (1) No fever for at least 3 days; (2) Improvement of splenomegaly measured by abdominal ultrasound; (3) At least a 50% decrease in soluble CD25, ferritin and HLH key cytokines including IFN-γ and IL-10; (4) At least one-fold increase of neutrophil count (G-CSF injection was allowed but should sustained improvement for at least 3 days); (5) No progression of other HLH markers. HLH improvement was defined as (13): (1) Body temperature <37.5 °C; (2) ≥ 3 HLH markers improved, but did not meet the aforementioned PR criteria. No Response (NR) was defined as failure to meet the criteria for HLH improvement. Progression defined as at least a 50% worsening in two or more signs or laboratory abnormalities (13). Refractory was defined as failure to achieve at least PR by week 2 of initial etoposide and MP-based therapy, or occurrence of disease progression or relapse during therapy (16, 17). Relapse was defined as the re-emergence of abnormalities in three or more symptoms and laboratory markers after having achieved CR. Event-Free Survival (EFS) is defined with the following events: no response, disease progression, relapse, or death from any cause. The evaluation period for EFS is measured from the initiation of treatment until the date of the first occurrence of any of these events (whichever occurs first) or the date of last follow-up.

### Outcomes

The primary outcome was 12-month overall survival rate (OS); The secondary outcome included 12-month event-free survival (EFS); early response and safety; dynamic changes in laboratory parameters; the cumulative doses of glucocorticoids and etoposide.

### Statistical analysis

Statistical analyses were performed using SPSS version 26.0. Categorical variables are presented as frequencies or percentages (n or %). Differences between groups for categorical variables were analyzed using the chi-square test or Fisher exact test, as appropriate. Differences in ordered categorical treatment response between groups were assessed using the Mann-Whitney U test. Continuous variables were assessed for normality. Normally distributed data are expressed as mean ± standard deviation (SD) and were compared between groups using the independent samples t-test. Non-normally distributed data are expressed as median and interquartile range (IQR) and were compared between groups using the Mann-Whitney U test. The dynamic changes in laboratory test results at multiple time points were compared using repeated measures analysis of variance. The Kaplan-Meier method was used to estimate the 12-month overall survival (OS) and event-free survival (EFS) rates for each group, presented with 95% confidence intervals (CI). Differences in survival curves between groups were evaluated using the log-rank test. A P-value < 0.05 was considered statistically significant.

## Results

### Characteristics of patients

A total of 67 patients with hemophagocytic lymphohistiocytosis (HLH) were included, with 40 assigned to Group C and 27 to Group T. Baseline demographic and clinical characteristics were comparable between the two groups (**Table 1**). The male-to-female ratio was 21:19 in Group C and 8:19 in Group T (P = 0.064). Median age was 62 months (range: 10–180m) in Group C and 54 months (range: 14–112m) in Group T (P = 0.378). All patients presented with fever > 38.5 °C. Splenomegaly was observed in 59% of Group C and 80% of Group T (P = 0.075), with no significant differences in other clinical features. Group T demonstrated significantly higher median neutrophil counts and plasma fibrinogen levels compared to Group C (P = 0.032 and P = 0.033, respectively), while alanine aminotransferase was lower in Group T (P = 0.039). Low natural killer cell activity was more frequent in Group C (94.7% vs. 50%, P < 0.001). Other laboratory parameters showed no significant differences. Secondary HLH was the predominant etiology in both groups (97.8% in Group C vs. 93.4% in Group T, P = 0.726). Among the 64 patients with secondary HLH, the specific etiologies were as follows: EBV-associated HLH (EBV-HLH) in 54 patients (84.4%), HLH associated with chronic active EBV infection (CAEBV-HLH) in 2 (3.1%), HLH of unknown origin in 8 (12.5%). The distribution of these etiologies did not differ significantly between Group T and Group C (P=0.217).

**Table 1:** Baseline characteristics of patients.

Patient subgroup	Total (n=67)	Group C (n=40)	Group T (n=27)	P value
General				
Gender(male/female),n,	29/38	21/19	8/19	0.064
Median age(range),m	60 (10,180)	62 (10,180)	54 (14,112)	0.378
Clinical parameters, %				
Fever(>38.5°C),%	100	100	100	
Splenomegaly,%	73.5	59	80	0.075
Hepatomegaly,%	89.7	84	80	0.943
Lymphadenopathy,%	79.4	68.1	80	0.273
Jaundice,%	22	22.7	16.6	0.532
CNS involvement,%	7.3	9	3.3	0.626
Laboratory findings				
White blood cells, ×10^9^/L	2.24(1.28,3.22)	2.34(1.38,4.47)	1.71(1.21,2.72)	0.065
Neutrophils, ×10^9^/L	0.66(0.43,0.91)	0.57(0.42,0.74)	0.88(0.48,1.39)	0.032
Platelets, ×10^9^/L	60(35,98)	68(42,94)	58(32,101)	0.539
Hemoglobin, g/L	91.84 ± 14.11	90.78 ± 15.12	93.41 ± 12.58	0.458
Fibrinogen, mg/dl	145(110,213)	131.5(103.7,193.7)	149(130,263)	0.033
Triglycerides, mmol/L	3.11(2.44,4.20)	3.36(2.74,4.33)	2.85(2.26,3.40)	0.095
AST, U/L	288.30(121.30,489.60)	354.40(132.95,499.55)	192.20(102.70,433.20)	0.154
ALT, U/L	206.30(84.80,371.60)	230.55(99.57,443.05)	150.80(56.50,281.50)	0.039
ALB,	33.35 ± 4.40	33.34 ± 4.47	33.38 ± 4.38	0.969
LDH,	949.00(709.00,1506.00)	958.00(742.25,1757.50)	899.00(530.00,1392.00)	0.225
Ferritin,	5423.20(1521.10,15000)	7114.00(1956.75,15000)	3561.65(1170.50,13480.95)	0.230
Soluble CD25,U/ml	9147.00(6102.50,15065.00)	10270.00(7142.00,16854.25)	8871.00(6076.00,13040.00)	0.368
Low NK cell activity, %	76.5	94.7	50	0.000
Hemophagocytosis, %	85.2	77.2	80	0.926
IFN-γ	1551.43(322.23,4359.67)	1008.21(253.75,3414.74)	3070.25(560.76,4642.60)	0.092
IL-10	98.04(33.29,367.51)	114.40(34.13,621.37)	79.52(33.29,276.10)	0.116
IL-6	31.22(13.44,71.04)	23.29(10.78,68.90)	32.58(16.37,71.04)	0.283
Etiology, n,%				
Primary HLH	3, 4.5	1, 2.2	2, 6.6	0.726
Secondary HLH	64, 96.5	39, 97.8	25, 93.4	
EBV-HLH	54, 84.4	31, 79.5	23, 92	0.217
CAEBV-HLH	2, 3.1	1, 2.6	1, 4	
unknown	8, 12.5	7, 17.9	1, 4	

### Response and clinical outcomes

#### Response to treatment

**Figure 1:**
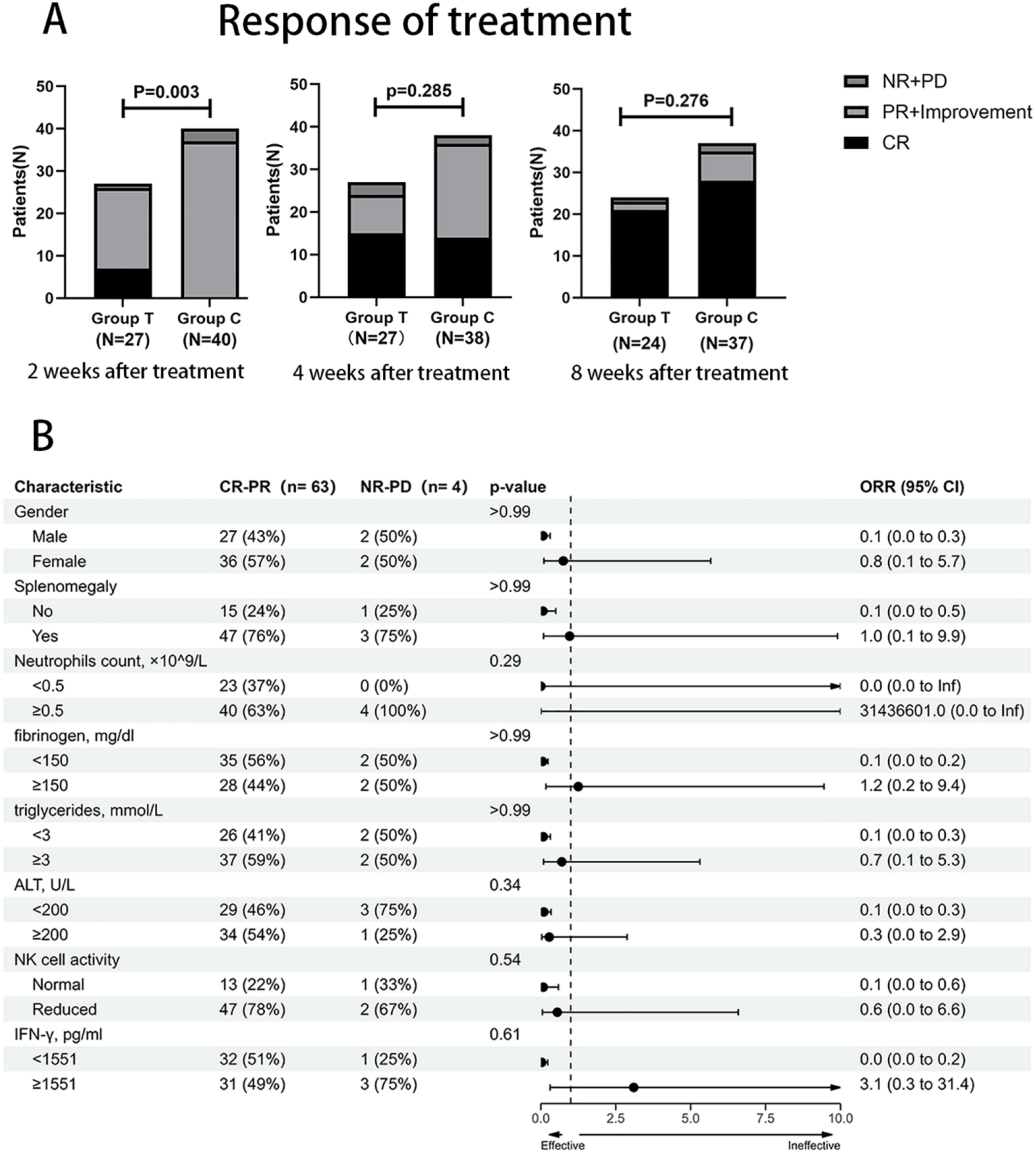
Comparison of treatment responses between the two groups and subgroup analysis. **(A)** Treatment responses at 2, 4, and 8 weeks post-treatment. CR, complete response; PR, partial response; Improvement, disease improvement; NR, no response; PD, progressive disease. **(B)** Subgroup analysis based on the 2-week treatment response.

Early response to treatment was markedly enhanced in Group T (**Figures 1A**; **Table 2**). At week 2, the complete remission (CR) rate was significantly higher in Group T than in Group C (25.9% vs. 0%; P = 0.001), though the overall response rate (ORR; comprising CR, partial remission, or clinical improvement) was comparable between groups (96.3% vs. 92.5%; P = 0.906). By week 4, CR rates were 55.5% (15/27) in Group T and 35.0% (14/40) in Group C (P = 0.096), while ORRs remained similar (88.9% vs. 90.0%; P = 1.000). At week 8, CR rates reached 78.0% (21/27) in Group T and 70.0% (28/40) in Group C (P = 0.481), with no significant difference in ORR (85.2% vs. 87.5%; P = 1.000). These results suggest that Group T led to more rapid achievement of CR without affecting ultimate ORR. Given the predominance of EBV-HLH, a subgroup analysis was performed. Within the EBV-HLH cohort (n=54), the CR rate at week 2 was significantly higher in Group T (30.4%) than in Group C (0%) (P = 0.002), recapitulating the early efficacy advantage observed in the overall population. Subgroup analysis based on baseline characteristics and the 2-week treatment response revealed that gender, splenomegaly, neutrophil count, fibrinogen, triglycerides, ALT, NK cell activity, and IFN-γ had no significant effect on the overall response rate (ORR) (**Figure 1B**). Subgroup analyses demonstrated that the advantage of a higher week-2 complete remission (CR) rate in Group T was statistically significant in the overall population. This advantage was particularly pronounced and significant in patients with diminished baseline NK-cell activity (P = 0.016), whereas no significant difference was observed in those with normal baseline NK-cell activity (P = 1.000). The benefit of a higher CR rate in Group T was consistently observed across subgroups defined by different baseline levels of absolute neutrophil count (ANC) and fibrinogen (all P values <0.05). A higher CR rate was also noted in patients with markedly elevated baseline alanine aminotransferase (ALT), although this difference did not reach statistical significance (P = 0.076) (**Supplementary Table S2**).

**Table 2:** Comparison of outcoms between Group T and Group C.

Outcome	Group T (n=27)	Group C(n=40)	P value
Infection,n,%	6, 22.2	10, 25	0.794
ORR at 2-weeks,n,%	26, 96.3	37, 92.5	0.906
CR rate at 2-weeks,n,%	7, 25.9	0, 0	0.001
ORR at 4-weeks,n,%	24, 88.9	36, 90.0	1
CR rate at 4-weeks,n,%	15, 55.5	14, 35.0	0.096
ORR at 8-weeks,n,%	23, 85.2	35, 87.5	1
CR rate at 8-weeks,n,%	21, 78.0	28, 70.0	0.481
Refractory/Relapse rate,n,%	7, 25.9	9, 22.5	0.747
OS at 12-months,n,%	26, 96.3	37, 92.5	0.643
EFS at 12-months,n,%	20, 74.1	31, 77.5	0.747

### Survival

**Figure 2:**
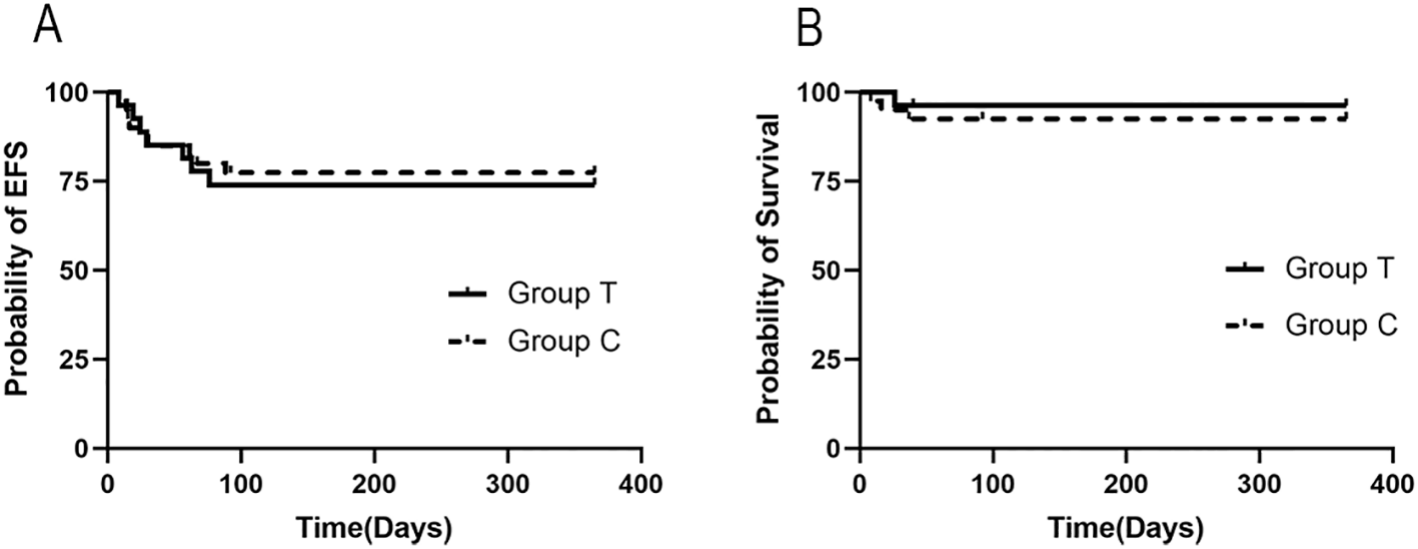
Kaplan-Meier analysis of 12-month EFS and OS between the two groups. **(A)** Comparison of the 12-month EFS in two group (Log-rank p = 0.766). **(B)** Comparison of the 12-month OS in two group (Log-rank p = 0.519).

Survival outcomes were analyzed at the 12-month time point. There were four deaths (one in Group T and three in Group C) during the follow-up, all due to disease progression and organ failure. The 12-month OS rate was 96.3% (95%CI: 89.2-100%) in Group T and 92.5% (95%CI: 84.3-100%) in Group C (p = 0.519 by log-rank test). The 12-month EFS rates were 74.1% (95%CI: 57.6-90.6%) and 77.5% (95%CI: 64.6-90.4%) for Group T and Group C, respectively (p = 0.766 by log-rank test). The survival curves for both OS and EFS remained largely overlapping through the first 50 days, with slight separation beyond that time point without any observed crossings (**Figure 2**). To assess the potential impact of the baseline NK cell activity imbalance, a Cox proportional hazards regression analysis was performed. After adjustment for baseline NK activity status, the hazard ratio (HR) for death in Group T compared to Group C was 1.05 (95% confidence interval [CI]: 0.09 to 12.39; P = 0.969). When adjusted for treatment group, baseline NK cell activity itself was not significantly associated with overall survival (OS) (P = 0.979). Furthermore, no statistically significant interaction was observed between treatment group and NK cell activity status (P = 0.992). Stratified Kaplan-Meier analysis with the Log-rank test corroborated these findings, indicating that baseline NK cell activity status was not a significant independent prognostic factor for 1-year OS (P = 0.785). Collectively, these results suggest that the treatment effect did not differ significantly between patients with low versus normal baseline NK activity. Similarly, analyses of baseline ANC, FIB, and ATL levels revealed no significant association with OS (all P > 0.05). Given the predominance of EBV-HLH, a subgroup analysis was performed. Within the EBV-HLH cohort (n=54), the 12-month cumulative OS rates were 95.7% (95% CI: 83.4, 99%) for Group T and 90.3% (95% CI: 76.8, 96.4%) for Group C (P = 0.459). The 12-month cumulative EFS rates were 78.3% (95% CI: 58.9, 89.5%) and 80.6% (95% CI: 62.5, 90.6%) for Group T and Group C (P = 0.825).

### Dynamic changes in laboratory parameters

**Figure 3:**
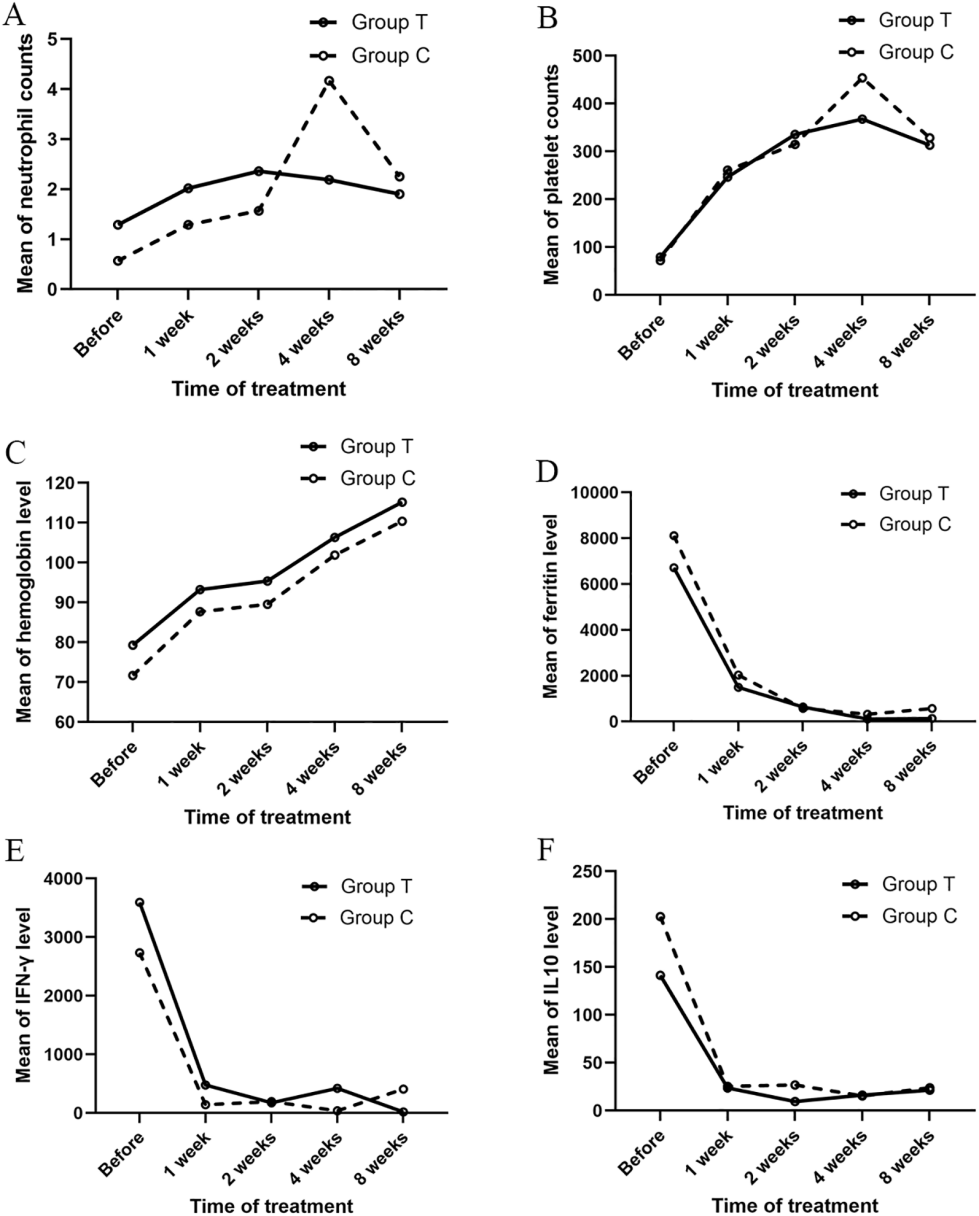
Dynamic changes in laboratory parameters between the two groups at baseline and at 1, 2, 4, and 8 weeks post-treatment. **(A)** Neutrophil counts. **(B)** Platelet counts. **(C)** Hemoglobin levels. **(D)** Serum ferritin levels. **(E)** IFN-γ levels. **(F)** IL-10 levels.

To systematically evaluate the impact of the two regimens on inflammatory activity and hematopoietic function, we serially monitored the levels of ANC, PLT, Hb, ferritin, IFN-γ, and IL-10 at baseline, 1, 2, 4, and 8 weeks. The results demonstrated a significant main effect of time for all measured parameters (P < 0.001) (**Supplementary Table S1**). A significant interaction effect was observed between the two groups for ANC. (P < 0.001, η² = 0.12). In Group T, the ANC exhibited an “early peak” pattern, rising to 2.36 ± 3.24 ×10^9^/L at week 2. Group C showed a delayed peak until week 4 (4.17 ± 2.95 ×10^9^/L), but remained stable in Group T (2.19 ± 1.49 × 10^9^/L), converging by week 8 (**Figure 3A**). Platelet counts increased markedly in both groups within one week, and remained within normal limits at all subsequent timepoints, with no significant interaction effect observed between the groups(P = 0.074) (**Figure 3B**). Hemoglobin levels increased gradually in both groups. Although Group T exhibited higher hemoglobin levels compared to Group C, no significant interaction effect was observed(P = 0.901) (**Figure 3C**). Serum ferritin levels in both groups declined within two weeks, no significant interaction effect was observed between the groups(P = 0.603) (**Figure 3D**). IFN-γ levels in both groups decreased rapidly within one week, no significant interaction effect was observed between the two groups(P = 0.404) (**Figure 3E**). IL-10 levels in both groups decreased rapidly within one week and were subsequently sustained at low levels thereafter, with no significant interaction effect observed between the groups (P=0.526) (**Figure 3F**).

### The cumulative doses of glucocorticoids and etoposide

**Figure 4:**
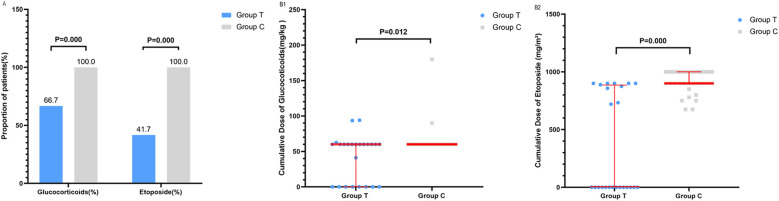
Comparison of glucocorticoid and etoposide utilization between treatment groups. The bar chart depicts the proportion of patients who received glucocorticoids or etoposide in Group T (n=24) and Group C (n=35). Data are presented as percentages. **(A)** Distribution of cumulative drug doses. **(B)** Individual data points represent the cumulative dose of glucocorticoids **(B1)** Group T median: 60mg/kg (IQR: 0, 60); Group C median: 60mg/kg (IQR:60, 60)) or etoposide **(B2)** Group T median: 0mg/m2 (IQR: 0, 885.5mg/m2); Group C median: 900mg/m2 (IQR:900, 1000)) for each patient in Group T (blue) and Group C (grey). The long red horizontal line indicates the median.

To assess the achievement of our treatment strategy aimed at minimizing cytotoxic exposure, we compared the cumulative doses of glucocorticoids and etoposide between the two groups. Since patients in the HLH-94 group(Group C) received dexamethasone as glucocorticoid, while those in the ruxolitinib-based regimen group (Group T) received methylprednisolone, dexamethasone doses were converted into methylprednisolone-equivalent doses to allow for comparison between the groups. For the comparative analysis of cumulative drug doses, data were included only for patients who completed the first 8 weeks of first-line therapy. Patients who died or required escalation to second-line treatment due to disease progression during this period were excluded from this analysis. Consequently, three patients from GroupT (one death, two progressions) and five from GroupC (three deaths, two progressions) were not included in the final statistical comparison. Among the 24 patients in the Group T, 8 received ruxolitinib monotherapy, 6 were treated with ruxolitinib plus glucocorticoids, and 10 received ruxolitinib combined with glucocorticoids and etoposide. All 35 patients in the Group C were treated with glucocorticoids combined with etoposide. The proportion of patients receiving glucocorticoids was significantly lower in Group T (66.7%, 16/24) than in Group C (100%, 35/35), with a statistically significant difference (P = 0.000). A significantly lower proportion of patients in Group T received etoposide compared to Group C (41.7% [10/24] vs. 100% [35/35]; P = 0.000) (**Figure 4A**). The patterns of glucocorticoid and etoposide use differed fundamentally between the protocols, reflecting the response-guided design of the ruxolitinib-based regimen. For glucocorticoids (expressed as methylprednisolone equivalent), the median cumulative dose of methylprednisolone in Group C was 60 mg/kg (IQR: 60, 60 mg/kg). In Group T, use was bimodal: patients with a favorable response to ruxolitinib monotherapy received none (0 mg/kg), while others received methylprednisolone therapy (≈60 mg/kg), resulting in a median of 60 mg/kg but a significantly different distribution (IQR: 0, 60 mg/kg; P = 0.012) (**Figure 4B1**). Similarly, for etoposide, all patients in Group C received it per protocol (median cumulative dose 900 mg/m²; IQR: 900, 1000 mg/m^2^). In Group T, it was avoided entirely in ruxolitinib and glucocorticoids responders and it used only for others, leading to a median cumulative dose of 0 mg/m^2^ (IQR: 0, 885.5 mg/m^2^; P < 0.001) (**Figure 4B2**). These differences highlight the reduced chemotherapy exposure facilitated by the ruxolitinib-based approach.

### Safety

This study compared the safety profiles of the two patient groups during the early phase of treatment. The incidence of secondary infections was 22% in Group T and 25% in Group C, with no statistically significant difference observed between the groups (P = 0.794). Furthermore, the proportions of patients who developed abnormalities in total bilirubin (TBil), aspartate aminotransferase (AST), alanine aminotransferase (ALT), and serum creatinine (Scr) levels during treatment were compared. No significant differences were found between the two groups in the incidence of abnormalities for any of these laboratory parameters (**Table 2**). No secondary malignancies, cardiovascular events, thromboembolic events, or gastrointestinal perforation events were observed during the follow-up period.

## Discussion

This study provides a comparative analysis of a ruxolitinib-based regimen versus the conventional HLH-94 protocol in pediatric HLH. The findings suggest that the ruxolitinib-based approach represents a promising therapeutic strategy, with its primary advantages manifesting as a more rapid induction of complete remission and a significant reduction in exposure to cytotoxic chemotherapy, while preserving high short-term survival with an acceptable safety profile.

In this study, the ruxolitinib-based regimen in pediatric HLH achieved a significantly higher complete response (CR) rate at the critical 2-week timepoint (25.9% vs. 0%, p = 0.001). This rapid response may carry important clinical implications, as prompt control of the life-threatening cytokine storm in HLH is essential for preventing organ damage and improving early outcomes. In the subgroup of patients with reduced baseline NK-cell activity, the higher complete remission (CR) rate in Group T at week 2 was more pronounced, but not in the subgroup of normal NK-cell activity, suggesting that the JAK/STAT pathway inhibitory mechanism of ruxolitinib may have an advantage in regulating immune dysfunction. But it also may be related to the small sample size and the fact that no patients in Group C achieved CR by 2-weeks, necessitating further validation with a larger sample. By week 8, both treatment groups exhibited comparable CR and overall response rates (ORR), suggesting high efficacy of both regimens. Both groups demonstrated high 12-month overall survival (OS) rates (Group T: 96.3% vs. Group C: 92.5%), with no statistically significant difference between them (P = 0.519). This may reflect comparable efficacy of both strategies in controlling the disease within this study cohort, but it could also be influenced by the limited sample size and follow-up duration. Our data suggest that a potential advantage of the investigational ruxolitinib-based regimen is that it can induce complete remission more rapidly while significantly reducing exposure to conventional chemotherapeutic agents, particularly etoposide, and still achieve a high short-term survival rate.

Ruxolitinib, a Janus kinase 1/2 inhibitor, can inhibit numerous cytokines and represents a promising therapeutic option for HLH patients (6, 18, 19). Previous studies have demonstrated the rapid onset of action of ruxolitinib in HLH, particularly in controlling inflammatory responses and cytokine release. A study in children with HLH showed that ruxolitinib, as first-line therapy, could induce rapid and sustained clinical remission in the majority of patients (11, 13). In Epstein-Barr virus-associated HLH (EBV-HLH), ruxolitinib-based regimens demonstrated efficacy comparable to traditional chemotherapy but with significantly reduced toxicity (20). For patients with primary HLH (pHLH), ruxolitinib-containing regimens not only effectively controlled the hyperinflammatory state but also improved pre-transplant disease stability and reduced the need for chemotherapeutic agents prior to hematopoietic stem cell transplantation (HSCT) (9). Furthermore, in children with autoimmune disease- or autoinflammatory disease-associated HLH (AD/AID-HLH), individualized ruxolitinib-based strategies have shown high response rates and a favorable safety profile, along with a marked reduction in the use of traditional cytotoxic drugs such as etoposide (10). Collectively, our findings, in conjunction with existing literature, provide preliminary support for the potential role of ruxolitinib but underscore the necessity for prospective, randomized head-to-head comparative trials to definitively establish its efficacy, safety, and positioning relative to current standard therapies.

The serial laboratory monitoring provides a biological rationale for the observed clinical efficacy. Laboratory monitoring revealed that both Group T and Group C exhibited rapid declines in ferritin, IFN-γ, and IL-10 levels, confirming the effective suppression of hyperinflammation by ruxolitinib (5, 21). Literature indicates that as a JAK1/2 inhibitor, ruxolitinib significantly reduces inflammatory markers such as ferritin and cytokine levels by blocking the signaling pathways of key cytokines including IFN-γ and IL-6, thereby mitigating the cytokine storm in HLH (6, 8). A notable difference was observed in the neutrophil recovery pattern: Group T demonstrated earlier and more stable recovery of absolute neutrophil count (ANC), whereas Group C showed delayed recovery with higher peak values. This suggests that ruxolitinib may promote more balanced immune reconstitution by modulating the JAK-STAT pathway, avoiding excessive inflammatory responses. Supported by existing studies, ruxolitinib not only inhibits IFN-γ signaling but also independently influences neutrophil activation and infiltration, thereby reducing tissue damage and cytokine release (6). The reduced exposure to etoposide in Group T also contributed to their earlier and more stable neutrophil recovery. Studies have demonstrated that the ruxolitinib-based treatment regimen is associated with a lower incidence of myelosuppression compared to chemotherapy-based regimens, with a significant reduction in the occurrence of grade 3 or higher myelosuppression (20). Furthermore, a rebound in ferritin and IFN-γ levels was observed in Group C at week 8, while these markers remained low in Group T, indicating that the response-based stratified treatment regimen with ruxolitinib provides more sustained control of inflammation. These findings suggest the role of ruxolitinib response-based stratified treatment strategy in HLH management, potentially supporting not only short-term inflammatory suppression but also long-term remission through modulated immune reconstitution (13), though this requires confirmation in longer-term studies.

A statistically significant difference in cumulative exposure to etoposide and glucocorticoids was observed between the two groups. The HLH-94 protocol (Group C) mandates the use of these agents, whereas the ruxolitinib-based strategy was prospectively designed to test whether upfront targeted therapy could safely enable a treatment de-escalation, reducing or eliminating the need for conventional cytotoxic drugs. Our results successfully validate this pre-specified design goal: In Group T, only 41.7% of patients required etoposide, while the median cumulative dose of etoposide was 0 mg/m^2^. It indicated that a majority of patients achieved excellent outcomes without any etoposide exposure. While glucocorticoid use was also significantly reduced. The clinical implications of successfully achieving this de-escalation are profound. Etoposide, a cornerstone of HLH-94, is associated with significant myelosuppression and a well-defined risk of secondary malignancies and treatment-related mortality (20, 21). In this study, we observed that Group T demonstrated superior hematological recovery compared to Group C. Platelet and hemoglobin levels showed early recovery starting from week 1 and were maintained within the normal range throughout the 8-week observation period. Notably, the absolute neutrophil count recovered earlier and more stably in Group T, indicating a preferential alleviation of HLH-associated myelosuppression, thereby achieving early hematopoietic recovery without worsening cytopenia. Additionally, no events of secondary malignancy, cardiovascular complications, thromboembolism, or gastrointestinal perforation were observed during the follow-up period. In fact, despite a substantial reduction in etoposide exposure, patients in Group T still achieved a high 12-month overall survival (OS) rate of 96.3%, along with a higher early complete remission (CR) rate. This demonstrates that ruxolitinib can offset the need for this cytotoxic agent in a considerable proportion of patients. This represents a potential paradigm shift towards a less toxic, targeted therapeutic approach, where the primary value proposition is the reduction of long-term toxicity risks without compromising survival. However, given the current relatively short follow-up duration and limited sample size, further extended monitoring and expanded studies with larger sample size are warranted to fully evaluate late effects, including the incidence of secondary neoplasms and other treatment-related sequelae, and to confirm the long-term benefit of this chemotherapy-reduced strategy.

It is imperative to underscore the preliminary nature of these conclusions. This study has several limitations. First, the retrospective, single-center, historical control design carries inherent risks of selection bias and unmeasured confounding, including potential time-related confounding as supportive care and management practices may have evolved between the treatment periods. Furthermore, notable baseline imbalances were observed (e.g., NK cell activity). Although we attempted to mitigate bias by applying consistent diagnostic criteria and conducting the study at a single center, and the potential confounding was explored and examined through subgroup analyses, the influence of these design-inherent limitations and baseline differences persists. Second, while the sample size was sufficient to demonstrate significant differences in early treatment response and cumulative drug exposure, it may be underpowered to detect subtle yet clinically meaningful differences in long-term survival outcomes or rare adverse events between the regimens. Relatedly, although a subgroup analysis was performed for the predominant EBV-HLH patients, the limited number of cases with other etiologies precluded a meaningful assessment of treatment efficacy across the broader spectrum of secondary HLH. Consequently, the generalizability of our findings may be primarily confined to the EBV-HLH subgroup. Finally, the 12-month follow-up period is relatively short. A longer observation time is necessary to fully evaluate the durability of remission, the long-term consequences of reduced chemotherapy and corticosteroid exposure, and the risk of late effects such as secondary malignancies. Future prospective, multi-center studies with larger sample sizes, stratified randomization, and longer follow-up are warranted to confirm these findings and to evaluate the efficacy of the ruxolitinib-based regimen across a more diverse etiological landscape.

## Conclusion

For pediatric HLH, the ruxolitinib-based regimen represents a viable and effective protocol. Its advantages include achieving complete remission more rapidly and significantly reducing the use of chemotherapeutic agents, particularly etoposide, while maintaining a high short-term survival rate and acceptable safety. However, due to the retrospective design, limited sample size, and relatively short follow-up of this study, these findings remain preliminary. Therefore, this work primarily provides a strong rationale for future prospective, randomized controlled trials, which are essential to confirm the long-term benefits and safety of this approach.

## Data Availability

The original contributions presented in the study are included in the article/**Supplementary Material**. Further inquiries can be directed to the corresponding author.

## References

[1] Trottestam H Horne A Aricò M Egeler RM Filipovich AH Gadner H . Chemoimmunotherapy for hemophagocytic lymphohistiocytosis: long-term results of the HLH-94 treatment protocol. Blood. (2011) 118:4577–84. doi: 10.1182/blood-2011-06-356261 21900192 PMC3208276

[2] Bergsten E Horne A Aricó M Astigarraga I Egeler RM Filipovich AH . Confirmed efficacy of etoposide and dexamethasone in HLH treatment: long-term results of the cooperative HLH-2004 study. Blood. (2017) 130:2728–38. doi: 10.1182/blood-2017-06-788349 28935695 PMC5785801

[3] Tahta N Acar SO Al IO Erdem M Karapınar TH Oymak Y . Efficacy of etoposide-based therapy in pediatric secondary hemophagocytic lymphohistiocytosis. Eur J Haematol. (2025) 116:319–25. doi: 10.1111/ejh.70082 41401815

[4] Meadows AT Friedman DL Neglia JP Mertens AC Donaldson SS Stovall M . Second neoplasms in survivors of childhood cancer: findings from the Childhood Cancer Survivor Study cohort. J Clin Oncol. (2009) 27:2356–62. doi: 10.1200/jco.2008.21.1920 19255307 PMC2738645

[5] Keenan C Nichols KE Albeituni S . Use of the JAK inhibitor ruxolitinib in the treatment of hemophagocytic lymphohistiocytosis. Front Immunol. (2021) 12:614704. doi: 10.3389/fimmu.2021.614704 33664745 PMC7923355

[6] Albeituni S Verbist KC Tedrick PE Tillman H Picarsic J Bassett R . Mechanisms of action of ruxolitinib in murine models of hemophagocytic lymphohistiocytosis. Blood. (2019) 134:147–59. doi: 10.1182/blood.2019000761 31015190 PMC6624972

[7] Das R Guan P Sprague L Verbist K Tedrick P An QA . Janus kinase inhibition lessens inflammation and ameliorates disease in murine models of hemophagocytic lymphohistiocytosis. Blood. (2016) 127:1666–75. doi: 10.1182/blood-2015-12-684399 26825707 PMC4817310

[8] Maschalidi S Sepulveda FE Garrigue A Fischer A Basile G . Therapeutic effect of JAK1/2 blockade on the manifestations of hemophagocytic lymphohistiocytosis in mice. Blood. (2016) 128:60–71. doi: 10.1182/blood-2016-02-700013 27222478

[9] Ge J Zhang Q Ma H Wang D Zhao Y Zhu T . Ruxolitinib-based regimen in children with primary hemophagocytic lymphohistiocytosis. Haematologica. (2024) 109:458–65. doi: 10.3324/haematol.2023.283478 37470145 PMC10828753

[10] Fang Z Wang D Ge J Zhao Y Lian H Ma H . Ruxolitinib-based regimen in children with autoimmune disease or autoinflammatory disease-related haemophagocytic lymphohistiocytosis. Br J Haematol. (2025) 206:215–23. doi: 10.1111/bjh.19803 39387140

[11] Zhang Q Wei A Ma H-H Zhang L Lian H-Y Wang D . A pilot study of ruxolitinib as a front-line therapy for 12 children with secondary hemophagocytic lymphohistiocytosis. Haematologica. (2021) 106:1892–901. doi: 10.3324/haematol.2020.253781 32732367 PMC8252948

[12] Chi Y Liu R Zhou Z-X Shi X-D Ding Y-C Li J-G . Ruxolitinib treatment permits lower cumulative glucocorticoid dosing in children with secondary hemophagocytic lymphohistiocytosis. Pediatr Rheumatol Online J. (2021) 19:49. doi: 10.1186/s12969-021-00534-0 33794928 PMC8015074

[13] Zhang Q Zhao Y-Z Ma H-H Wang D Cui L Li W-J . A study of ruxolitinib response-based stratified treatment for pediatric hemophagocytic lymphohistiocytosis. Blood. (2022) 139:3493–504. doi: 10.1182/blood.2021014860 35344583

[14] Henter J-I Horne A Aricó M Egeler RM Filipovich AH Imashuku S . HLH-2004: Diagnostic and therapeutic guidelines for hemophagocytic lymphohistiocytosis. Pediatr Blood Cancer. (2007) 48:124–31. doi: 10.1002/pbc.21039 16937360

[15] Zhang J-R Liang X-L Jin R Lu G . HLH-2004 protocol: diagnostic and therapeutic guidelines for childhood hemophagocytic lymphohistiocytosis. Zhongguo Dang Dai Er Ke Za Zhi = Chin J Contemp Pediatr. (2013) 15(8):686–8. 23965886

[16] Wang Y Huang W Hu L Cen X Li L Wang J . Multicenter study of combination DEP regimen as a salvage therapy for adult refractory hemophagocytic lymphohistiocytosis. Blood. (2015) 126:2186–92. doi: 10.1182/blood-2015-05-644914 26289641 PMC4635114

[17] Wei A Ma HH Zhang LP Lian HY Du JY Wang D . Ruxolitinib combined with liposomal doxorubicin, etoposide, methylprednisolone+/-PEG-asparaginase in treatment of relapsed/refractory pediatric hemophagocytic lymphohistiocytosis. Zhonghua Yi Xue Za Zhi. (2022) 102:2167–72. doi: 10.3760/cma.j.cn112137-20211224-02888 35872580

[18] Broglie L Pommert L Rao S Thakar M Phelan R Margolis D . Ruxolitinib for treatment of refractory hemophagocytic lymphohistiocytosis. Blood Adv. (2017) 1:1533–6. doi: 10.1182/bloodadvances.2017007526 29296794 PMC5728466

[19] Ahmed A Merrill SA Alsawah F Bockenstedt P Campagnaro E Devata S . Ruxolitinib in adult patients with secondary haemophagocytic lymphohistiocytosis: an open-label, single-centre, pilot trial. Lancet Haematol. (2019) 6:e630–7. doi: 10.1016/s2352-3026(19)30156-5 31537486 PMC8054981

[20] Wang W Zhao Y Ge J Fang Z Zhou C Wang D . Efficacy and safety of ruxolitinib-based regimen in the treatment of paediatric Epstein-Barr virus-associated haemophagocytic lymphohistiocytosis. Br J Haematol. (2025) 00:1–9. doi: 10.1111/bjh.20264 40665481

[21] Jianguo L Zhixuan Z Rong L Xiaodong S . Ruxolitinib in alleviating the cytokine storm of hemophagocytic lymphohistiocytosis. Pediatrics. (2020) 146:e20191301. doi: 10.1542/peds.2019-1301 32680878

